# A survey of the experience of living with dementia in a dementia-friendly community

**DOI:** 10.1177/1471301220965552

**Published:** 2020-10-08

**Authors:** Nicole Darlington, Antony Arthur, Michael Woodward, Stefanie Buckner, Anne Killett, Louise Lafortune, Elspeth Mathie, Andrea Mayrhofer, John Thurman, Claire Goodman

**Affiliations:** Centre for Research in Public Health and Community Care, 3769 University of Hertfordshire, Hertfordshire, UK; Faculty of Medicine and Health Sciences, 83726 University of East Anglia, Norwich, UK; Cambridge Institute of Public Health, 2152 University of Cambridge, Cambridge, UK; Faculty of Medicine and Health Sciences, 83726 University of East Anglia, Norwich, UK; Cambridge Institute of Public Health, 2152 University of Cambridge, Cambridge, UK; Centre for Research in Public Health and Community Care, 3769 University of Hertfordshire, Hertfordshire, UK; Centre for Research in Public Health and Community Care, 3769 University of Hertfordshire, Hertfordshire, UK; Faculty of Medicine and Health Sciences, 83726 University of East Anglia, Norwich, UK; Centre for Research in Public Health and Community Care, 3769 University of Hertfordshire, Hertfordshire, UK

**Keywords:** dementia, dementia friendly community, survey, questionnaire, England, awareness

## Abstract

Dementia-friendly communities (DFCs) are one way in which people living with dementia can be supported to be active, engaged and valued citizens. Quantitative evaluations of the experiences of those with dementia living within these communities are scarce. This article reports findings from a survey of people living with dementia on their experience of living in a DFC. Two-hundred and forty people living with dementia completed a cross-sectional survey in six DFCs across England. Around half of respondents reported they were aware they were living in a DFC. Being aware of living in a DFC was associated with taking part in leisure activities (*p* = 0.042), community centre attendance (*p* = 0.009), being involved in organised activities or groups (*p* < 0.001), feeling understood (*p* = 0.008), and feeling valued for their own contributions to the community (*p* = 0.002). This study illustrates the contribution that surveys can make in facilitating the expression of views and experiences of people living with dementia. Awareness of living within a DFC is associated with greater involvement in, and belonging to, the wider community.

## Introduction

In England, dementia-friendly communities (DFCs) are most often defined by a physical location (e.g., town, city and village). They commonly bring together local organisations and services to raise awareness, engage communities, challenge stigma, and normalise the experience of living with dementia (Buckner et al., 2019). A key aspiration of a DFC is to become a place where people living with dementia are included and empowered and not limited by the attitudes of other people, the local infrastructure, and how services are organised. Ninety percent of OECD (Organisation for Economic Co-operation and Development, 2018) countries support DFC initiatives to enable people affected by dementia to live well within their local communities. DFCs are designed to support people living with dementia to be active, engaged and valued citizens (Alzheimer’s Society, 2020).

Previous work has demonstrated the widespread reach of DFCs (Woodward et al., 2019) with upwards of 346 DFCs recognised in England (Dementia Friends, 2019). However, because they operate in variable and specific contexts and their work is widespread, the impact of a DFC upon people affected by dementia is difficult to judge. There are calls for more quantitative data to underpin the evidence base that informs the evolution of new DFCs and the development of existing ones (Herbert & Scales, 2019). Evaluative research to date is predominantly descriptive and restricted to qualitative data (Herbert & Scales, 2019; Lin & Lewis, 2015).

In the last decade, researchers have taken more steps to include people with dementia in quantitative research by addressing perceived difficulties in consent, capacity, and communication (Allen et al., 2017; Brooks et al., 2017). Research that only collects proxy accounts, such as those from family members and caregivers, to capture the experience of people living with dementia is not always reliable (Lepore et al., 2017; Rivett, 2017). In 2013, Alzheimer’s Society (UK) invited people living with dementia to complete a survey on their experiences of DFCs. A self-selecting sample of 500 people responded, with 17% completing this on their own behalf and 81% completing it with help from another person. Fewer than half thought the local environment enabled them to live well with dementia, and fewer than half felt part of their community (Green & Lakey, 2013).

This article reports on a survey of people living with dementia who reside in six DFCs but who were not directly involved in the DFC organisation or work (e.g., sitting on steering groups and attending DFC-specific activities). The survey only included the views and experiences of people living with dementia and explored the impact of living in a community aiming to become dementia-friendly. This survey aimed to answer the following questions: (1) Are people living with dementia aware of their local DFC? (2) Does awareness of a local DFC affect their experience of living with dementia? (3) What would help people living with dementia to live well in their local community? (4) Can survey methodology be used successfully among people living with dementia?

## Methods

### Sites

This study is part of a three-year NIHR Policy Research Programme Study (The DEMCOM study). In addition to a survey, this mixed-method study involved a scoping review (Buckner et al., 2019), a mapping of provision against dementia prevalence (Woodward et al., 2019), case studies and documentary analysis of how DFCs have become established in England.

The six sites selected were diverse in terms of their location across England, the populations they served and the length of time they had been in existence for. The six study sites were all recognised by Alzheimer’s Society as DFCs and had been in operation for over two years. Two of the six sites were considered to be ethnically diverse. All six sites were based in England: three were cities, one a large town, one consisted of a town ‘hub’ with smaller satellite DFCs and one a small market town.

### Instruments

A postal questionnaire with eight questions was developed and piloted. It drew on previous questionnaires with people living with dementia (Carter & Rigby 2017; Hampshire County Council, Innovations in Dementia & Local Government Association (2012)) and work by Buckner et al. (2019). The questionnaire was structured to cover the respondent’s awareness of DFC activity and services, activities and environments they enjoyed, and their day-to-day involvement in the local community. There was also space for free text responses and comments. The questionnaire had seven iterations and was reviewed by six people who had direct experience of living with and being affected by dementia. As a result of this input, the questionnaire was shorter and the language was more accessible (see Supplemental material). The focus of the survey was to measure the experiences of people living with dementia in a DFC. We believed that a survey might be acceptable to this population as it was potentially less burdensome than undertaking more in-depth interviews, and offered the opportunity to gain a broader picture from a larger sample than restricting our study to qualitative methods.

### Sample and recruitment

The survey sample included all people living with dementia who were not directly involved in the running or planning of their local DFC. To maximise recruitment opportunities, a variety of methods were used to reach potential participants. These varied between sites. Questionnaires were distributed in partnership with local Clinical Research Networks, via Join Dementia Research (www.joindementiaresearch.nihr.ac.uk), through research databases held at memory clinics, and via primary care practices. In one site, where Alzheimer’s Society was represented but not involved in the organisation of the DFC, the surveys were distributed via the local Alzheimer’s Society branch. The number of questionnaires to be distributed was negotiated with individual organisations and determined on the basis of their existing workload.

People living with dementia had the choice of completing the questionnaire over the phone or on paper (with a prepaid return envelope). They were encouraged to either do this on their own or ask someone to support them and report their answers on their behalf. Participants were asked to respond in a way that reflected their own, rather than others’, views and experiences of living with dementia. Consent to participate was assumed through the act of completing and returning the questionnaire, and it was stressed that this was entirely voluntary. Where questionnaires were administered over the telephone, the staff member established at the outset that the respondent was happy to answer the questions. In this case, the person administering the questionnaire was trained to remain alert as to whether the participant was becoming tired or distressed in which case the telephone call was drawn to a close. A separate consent form was not included in order to avoid overburdening prospective participants and to protect their anonymity.

Data collection took place over a year between March 2018 and March 2019.

### Data analysis

Survey data were analysed using SPSS version 25 (IBM Corp. Released, 2017). Response rate and participants characteristics were summarised descriptively. Cross-tabulation with chi-squared tests were used to compare respondents’ awareness of living in a DFC (yes/no) and a series of binary variables related to statements about their involvement in activities and experiences of living in a DFC. Where expected cell counts were fewer than five, Fisher’s exact tests were used (Fisher, 1992).

## Results

Recruitment methods and response rates varied by site. In total, 244 participants were recruited to the study, with a response rate of 43%. Four questionnaires were excluded due to missing or incomplete data (*n* = 3) or not being filled out on behalf of somebody with dementia (*n* = 1), leaving the total number as 240.

The respondents included 106 males (46%) and 126 females (54%). Most were aged 75 years or over (*n* = 159, 67%) and identified as being in the ‘early’ stages of dementia (*n* = 127, 57%). Ten participants (5%) were from Black, Asian and minority ethnic backgrounds (see Table 1).

**Table 1.** Description of sample (*N* = 240).

		*N* (%)			*N* (%)
Site	A	73 (30.4)	Race/ethnicity	White British/white other	191 (95.0)
	B	22 (9.2)		Black, Asian and minority ethnic	10 (5.0)
	C	9 (3.8)		NR	39
	D	78 (32.5)	Stage of dementia	Early	127 (57.0)
	E	35 (14.6)		Middle	72 (32.3)
	F	23 (9.6)		Advanced	24 (10.8)
Gender	Male	106 (45.7)		NR	17
	Female	126 (54.3)	Residential status	Lives alone	59 (25.3)
	NR^a^	8		Lives with another person	174 (74.7)
Age (years)	<64	18 (7.8)		NR	7
	65–74	58 (25.1)	Completed questionnaire unaided	Yes	51 (22.0)
	75–84	104 (45.0)		No	181 (78.0)
	85+	51 (22.1)		NR	8
	NR	9			

^a^NR = not reported.

Across the six sites, there was almost an equal split between those who knew about the DFC status of their community and those who did not, with a slight majority (*n* = 124; 52%) of participants unaware that their community was working towards being dementia-friendly.

Awareness of the local DFC was greater among those reporting taking part in leisure activities (*p* = 0.042) and those attending community centres (*p* = 0.009, see Table 2). Awareness of the local DFC presence was positively associated with attendance at organised activities that were dementia specific, such as ‘Singing For The Brain’® and dementia cafés (*p* < 0.001). Although the number of participants who reported being part of service user groups (*n* = 16) or research activities (*n* = 20) was relatively low, these respondents were proportionately far more likely to be aware of their DFC than those who were not. Awareness of a DFC was also associated with people living with dementia saying that they felt they were understood (*p* = 0.008) and valued (*p* = 0.002) in their local community. Dementia-friendly community awareness did not differ according to whether respondents felt safe or had stopped doing things. Seven of the ten participants from Black, Asian and minority ethnic backgrounds reported not being aware of their local DFC.

**Table 2.** Activities and perceptions of survey responders by awareness of dementia-friendly communities (*N* = 240).

			Awareness of DFC^a^		
			Yes, *n* (%)	No, *n* (%)	p-value
Activity †	Go to work	Yes	3 (42.9)	4 (57.1)	
		No	110 (47.8)	120 (52.2)	0.55
	Meet with friends/family	Yes	83 (48.5)	88 (51.5)	
		No	30 (45.5)	36 (54.5)	0.67
	Leisure activities	Yes	40 (58.0)	29 (42.0)	
		No	73 (43.5)	95 (56.5)	0.042*
	Go out to pubs/cafés	Yes	67 (49.3)	69 (50.7)	
		No	46 (45.5)	55 (54.5)	0.57
	Shopping and errands	Yes	77 (50.3)	76 (49.7)	
		No	36 (42.9)	48 (57.1)	0.27
	Go to community centre	Yes	31 (64.6)	17 (35.4)	
		No	82 (43.4)	107 (56.6)	0.009*
	Use public transport	Yes	46 (50.0)	46 (50.0)	
		No	67 (46.2)	78 (53.8)	0.57
	Go for a walk	Yes	59 (48.4)	63 (51.6)	
		No	54 (47.0)	61 (53.0)	0.83
	Religious activities	Yes	24 (58.5)	17 (41.5)	
		No	89 (45.4)	107 (54.6)	0.13
	Other	Yes	38 (49.4)	39 (50.6)	
		No	75 (46.9)	85 (53.1)	0.72
Involvement in organised activities/groups	None	Yes	41 (35.0)	76 (65.0)	
		No	68 (61.3)	43 (38.7)	<0.001*
	Dementia-specific activities	Yes	47 (72.3)	18 (27.7)	
		No	62 (38.3)	100 (61.7)	<0.001*
	Dementia support group	Yes	19 (63.3)	11 (36.7)	
		No	90 (45.7)	107 (54.3)	0.07
	Dementia action alliance	Yes	1 (50.0)	1 (50.0)	
		No	108 (47.8)	118 (52.2)	>0.99
	Service user group	Yes	13 (81.3)	3 (18.8)	
		No	96 (45.3)	116 (54.7)	0.008*
	Research group	Yes	14 (70.0)	6 (30.0)	
		No	95 (45.7)	113 (54.3)	0.038*
	Other	Yes	26 (56.5)	20 (43.5)	
		No	83 (45.6)	99 (54.4)	0.19
Stopped doing things		Yes	67 (45.6)	80 (54.4)	
		No/DK	44 (52.4)	40 (47.6)	0.32
Feel safe in their community		Yes	50 (53.8)	43 (46.2)	
		No/DK	60 (43.2)	79 (56.8)	0.11
Feel well understood		Yes	31 (64.6)	17 (35.4)	
		No/DK	77 (43.0)	102 (57.0)	0.008*
Valued for own contributions		Yes	32 (66.7)	16 (33.3)	
		No/DK	75 (41.9)	104 (58.1)	0.002*

DFC = dementia-friendly community; DK= do not know. *p < 0.05, †Participants could pick more than one option for this question.

^a^4 missing values for this question.

In addition to the nine activities pre-specified in the questionnaire, 78 respondents reported participating in a further 95 ‘other’ activities. Of these, 57 involved people living with dementia going out into the community. Fifteen respondents took park in group activities such as singing, gardening, bingo and a film club. Ten people were involved in sporting activities (cycling, walking and watching football), with only one sporting activity relying on the involvement of other people (dancing). Six participants reported going out to local shops and eateries. Two people took the opportunity to report that they did nothing, and others mentioned non-specific activities such as spending time with family. One hundred and forty-eight (61%) respondents said that they had stopped one or more activities because of dementia. Of these, driving was the most frequently reported activity no longer undertaken (32%), with 21% stating that they no longer went out alone.

Participants were asked “What would most help you to live well with dementia in [site]?” and to select one of four pre-specified options or ‘other’ (see Supplemental material). Thirty-three participants ticked more than one option for this question. Of the 240 participants in this study, 102 (43%) participants responded that *more public understanding of what it is like to live with dementia* would help them to live well in their community, 52 (22%) selected *extra support in public spaces*, 49 (20%) selected *larger choice of enjoyable activities* and 22 (9%) selected *better public transport*. Some participants left this question blank (*n* = 42, 17.5%).

When these results were split by awareness of living in a DFC, and when respondents who ticked more than one option (*n* = 33) were excluded, more public understanding of dementia was sought by those who were aware of their local DFC (*n* = 43, 44.3%) than those who were not (*n* = 32, 29.9%, *p* = 0.030). Similar results were also found when the 33 excluded individuals were included (aware: *n* = 55, 48.7%, unaware: *n* = 46, 37.1%).

A fifth of respondents (*n* = 42) offered additional suggestions as to what would improve their experience of living with dementia. Many of them were alternative expressions of the need to be free from stigma, to maintain their independence, and to feel they had a purpose and could still contribute (e.g., to continue driving and to teach others). There were a small number (*n* = 4) of responses that indicated the respondents did not personally want anything or did not want attention drawn to their dementia.

## Discussion

This article reports findings from a survey of people living with dementia regarding their experience of living in a DFC. It illustrates the contribution that surveys can make in facilitating the expression of views and experiences of people living with dementia through different pathways to explore the reach and impact of dementia-friendly initiatives. The survey found that if people living with dementia were aware of their local DFC, they were more likely to have higher expectations for positive public attitudes and participate in a range of local activities.

The need for more accessible and reliable transport was highlighted by 22 survey participants. This appeared linked to the recent experience of giving up driving. It is perhaps a reflection of the sample where 57% described themselves as living with the early stages of dementia, a time where driving ability and performance can be queried and re-tested (Alzheimer’s Society, 2016; Duchek et al., 2003). Other studies have emphasised the need for accessible transport for people living with dementia as an important focus for the work of DFCs (Crampton & Eley, 2013).

Awareness of living in a DFC was positively associated with wanting more public understanding. This can be interpreted in two ways. It could suggest that people living with dementia with higher expectations for positive public attitudes will find their DFC. Alternatively, and more positively, it could mean that experience of benefiting from a DFC changes expectation and a recognition that people’s attitudes can be more disabling than the symptoms of living with dementia. Phillipson et al. (2019) found that directly involving people with dementia in local awareness campaigns was an effective way to promote positive attitudes, reduce dementia-related stigma and improve public understanding in their local community.

In this study, five per cent of the sample were from Black, Asian and minority ethnic communities. This is higher than other large surveys of DFCs (e.g., Carter & Rigby, 2017). Although this subsample is too small to make any statistical inference, seven of the ten people living with dementia from Black, Asian and minority ethnic communities were not aware of their local DFC. This raises important questions about culturally sensitive initiatives, especially as two of the sites were ethnically diverse. One participant in particular answered positively about their local community but took the time to write on the survey that their answers only applied to “those who speak my language”. With the number of people with dementia from Black, Asian and minority ethnic groups expected to rise significantly as this population ages (All-Party Parliamentary Group on Dementia, 2013), and in addition to the barriers often experienced by this group in accessing post-diagnostic dementia care and support, there is a growing need for DFCs to develop methods of engaging with the diversity of people living with dementia (Nielsen et al., 2019).

Many studies recognise the benefits of getting out and engaging with the local community (Clark et al., 2018; Duggan et al., 2008). Similar to Carter & Rigby (2017), the results from our survey confirm that many people living with dementia still enjoy and engage in activities that require leaving the house. Not surprisingly, attendance at ‘dementia-specific’ activities such as Singing For The Brain® and dementia cafes, dementia-specific research and service user groups was positively associated with DFC awareness. Outward facing activities that target people living with dementia were valued. Qualitative data from the broader evaluation of the six sites, reported elsewhere (Goodman et al., 2020), found that when these activities were located within mainstream services and organisations (e.g., shopping centres, eateries, leisure and sports centres), they had the potential to bridge the experience of living with dementia with everyday life, providing continuity and normalising the needs of people living with dementia. In contrast, where these activities were separate and standalone, whilst equally appreciated, they appeared to have less impact on the wider community and were harder to sustain.

People living with dementia describe the importance of living in safe and accessible environments, and this being a measure of living well (Harding et al., 2019; Innovations in Dementia, 2011). We found no evidence that for people living with dementia, awareness of the local DFC was associated with feeling safe in the community. This is not to say that DFCs make no difference to safety, rather that the idea of feeling safe is more personal and complex than whether a person is aware of their DFC. Førsund et al. (2018) document how some people living with dementia found that activities in well-known places allowed them to feel safe, yet that ultimately people living with dementia felt most safe in their own homes. This raises the question as to how DFCs reach out to those who are predominantly housebound. Our wider national evaluation found that much of DFC work is focused on bringing people into activities (Goodman et al., 2020).

It is important to note that not all people living with dementia chose to engage with their local DFC; a small number even actively avoided it. Reasons included the physical environment, and not wanting to be identified as having dementia when in public. Opinions differ as to whether DFCs are helpful to those with dementia or patronising and highlighting their diagnosis (Rahman & Swaffer, 2018). While the ultimate aim of DFCs is to foster inclusion and acceptance, the results from this survey are an important reminder of this alternative narrative to DFCs and that one size does not necessarily fit all.

### Strengths and limitations

The survey was developed in partnership with patient and public involvement representatives from the DEMCOM evaluation framework. Although we did not undertake a formal validation process, it provided a useful insight into the views and experiences of people living with dementia in DFCs, and other large surveys using unvalidated surveys have been known to contribute to policy and practice (Green & Lakey, 2013, Carter & Rigby, 2017). There are calls for more quantitative approaches to evaluating DFCs and developing methods to measure their impact (Herbert & Scales, 2019). This study demonstrates that survey methods are possible; however, there are challenges to overcome to ensure that the voices of those with advanced dementia are heard in studies of this kind—only 10.8% of our survey participants had advance dementia. Moreover, 78% of the participants in this survey completed the questionnaire with support from another person. Although the researchers cannot be certain that the supporter did not influence the participant’s answers, allowing this support enabled the researchers to capture the voice of a large percentage of people living with dementia who would otherwise be excluded from sharing their experience. The results from this study complement the work of other studies that have developed outcome measures with people living with dementia (e.g., Harding et al., 2019) and resonates with their findings.

Sampling methods differed between the different sites, with one of the sites only yielding nine respondents. This, however, was expected due to the differences in sizes of the sites. Sampling methods were aimed at reaching people living with dementia who were unknown to, or not actively involved in, their local DFC. Participants were, however, in contact with services and had a known diagnosis of dementia. It is important to appreciate that there are large numbers of individuals in England without a diagnosis who are not in contact with services, and the results from this study cannot capture the experience of these individuals (Green & Lakey, 2013). This study does, however, emphasise the value of working closely with services that routinely support people living with dementia (e.g., memory clinics and GP practices) as a method of systematically communicating and capturing their experiences. We cannot infer causality due to the nature of the study design, and DFC awareness may come as the result of an already active and engaged life rather than the trigger.

## Conclusion

When evaluating the impact and reach of DFCs, it is important to actively involve and seek out people living with dementia who are the target beneficiaries. This survey provided evidence from people living with dementia who were not linked to the organisation of the DFC. It found that when people were aware of their DFC they were more likely to be active and to have higher expectations with regard to living in a community free from stigma. Communities should be concerned that many people living with dementia documented considerable losses and a shrinking of their world. These survey findings underline the need to build in methods of review and audit as an integral part of dementia-friendly initiatives to ensure that there is a match between the use of resources and the experiences and priorities of the local population.

## Supplemental Material

Supplemental_material – Supplemental Material for A survey of the experience of living with dementia in a dementia-friendly community Click here for additional data file. Supplemental Material, Supplemental_material for A survey of the experience of living with dementia in a dementia-friendly community by Nicole Darlington, Antony Arthur, Michael Woodward, Stefanie Buckner, Anne Killett, Louise Lafortune, Elspeth Mathie, Andrea Mayrhofer, John Thurman and Claire Goodman in Dementia
