# Supplementary material for: A survey of the experience of living with dementia in a dementia-friendly community
Source: Dementia (London). 2020 Oct 8;20(5):1711–22. doi: 10.1177/1471301220965552 (PMC8216308; doi:10.1177/1471301220965552)
Supplement: Supplemental_material – Supplemental Material for A survey of the experience of living with dementia in a dementia-friendly community [file Supplemental_material.docx]

# APPENDIX: Questionnaire

| **National Evaluation of Dementia Friendly Communities**  We want to ask you some questions about living with dementia in [site]  There are 8 questions on 3 pages. You do not need to answer all of them. Any information you give is very valuable to us.  We will keep your answers confidential. If you have any questions, please call [name] (DEMCOM researcher) on **[number here]** or email **[email address here]**  Please return your completed questionnaire in the prepaid envelope.  **Thank you for sharing your views.** |
| --- |


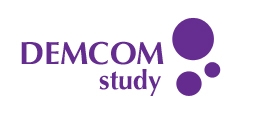


**1. Which of the following best describes you?**

I am filling in this questionnaire on my own

Someone else is helping me to fill in this questionnaire

**2. Are you aware that [site] is trying to become a ‘Dementia Friendly Community?’**

Yes  No

**3. What sort of things do you do now in [site]?** (Tick all that apply)

Go to work

Meet with friends/family in the home

Leisure activities (e.g. cinema, clubs, sports)

Go out to pubs/restaurants/cafés

Shopping and errands (e.g. banking)

Go to a community centre

Use public transport

Go for walk

Attend religious services

Other (Please say)

**4. Have you stopped doing anything because of dementia?**

No

Yes (please say)

**5. What would most help you to live well with dementia in [site]?**

(Tick one only)

Members of the public understanding what it is like to live with dementia

Extra support in public places (e.g. shops, cinema, sports centres)

Larger choice of enjoyable activities

Better public transport

Other (Please say)

Please explain your answer

**6. How did you find out about services and activities around dementia in [site]?**

(Tick all that apply)

Memory Clinic

GP practice

Alzheimer’s Society

The Council

Friends and family

Internet

Other (Please say)

**7. Which dementia groups in [site] are you part of?**

Activities for people living with dementia (e.g. Singing For the Brain, Dementia café)

Dementia support group

Dementia Action Alliance

Service user group

Dementia research group

Other (please say) _______________________________________

None

**8. Do you agree with these statements?** (Please tick)

a) People living with dementia can feel safe when they go out in [site]

Yes  No  Don’t know

b) What it means to live with dementia is well understood in [site]

Yes  No  Don’t know

c) People living with dementia are valued for their contributions in [site]

Yes  No  Don’t know

**About yourself**

**I am**

Under 55  55-64  65-74  75-84  85+

**I am**

Male Female Prefer not to say

**How would you describe your ethnic background** (e.g. White British, White other, Asian, African, Chinese)

_____________________________________________________

**I am in the**

Early stages of dementia

Middle stages of dementia

More advanced stages of dementia

**I normally live**

On my own

With someone who supports/cares for me

With someone who is not my carer/person who supports me

In a care home

Other (please say)

**What is the first part of your post code** (e.g. SG1)

_____
